# Report on the Sanitary Condition of the City of Chicago, and the Prevalence of Diseases from October 1st, 1867, to April 1st, 1868

**Published:** 1868-05

**Authors:** N. S. Davis

**Affiliations:** Member of Sanitary Committee


### CHICAGO MEDICAL EXAMINER.

N. S. DAVIS, M.D., Editor.

VOL. IX. MAY, 1868. NO. 5.

### Original Contributions.

### ARTICLE XIV.

### REPORT ON THE SANITARY CONDITION OF THE CITY OF CHICAGO, AND THE PREVALENCE OF DISEASES FROM OCTOBER 1st, 1867, TO APRIL 1st, 1868.

By N. S. DAVIS, M.D., Member of Sanitary Committee.

Since the date of my last report to this Society, no diseases of a strictly epidemic character have been prevalent in this city. The meteorological conditions have been less variable, the extremes of heat and cold less, and the wet or rainy days less than the average of winter and spring months in this locality.

The small-pox has continued to find victims amongst our heterogeneous population, in numbers sufficient to materially increase the aggregate mortality. The deaths from this disease were, in October, 20; November, 18; January, 39; February, 33; March, 37.

In the efforts made by those acting under the direction of the Board of Health and others, to stop the further prevalence of this disease by vaccination, two errors appear to have been committed in some instances. The first was, in not exercising due care in the selection and preservation of the vaccine used. It is certain that an unusually large number of cases have been followed by bad results, such as large phagadænic ulcers, cellular abscesses, and persistent eruptions, both pustular and vesicular. In two children who came under my care, the vaccine pustules had been accompanied by the most intense inflammation, ending in gangrene, and the formation of foul ulcers extending half the circumference of the arm, with a very serious degree of constitutional disturbance. Four others that I saw, had very extensive ulcerations, but without gangrene. A few days since, a gentleman, past the middle period of life, called at my office to consult me about his arm. He had been vaccinated in early life, the cicatrix of which was still visible. Five or six weeks since, he had been re-vaccinated. It produced a moderate-sized pustule, which matured in the usual time and cicatrized, leaving nothing unusual in the scar. In about one week after the vaccine sore had healed, an eruption began to appear on the arm, in the vicinity of the vaccine scar, and has continued to the present time. The eruption presents two forms: one is pustular, presenting much the shape and size of the larger pustules of acne, and very painful; the other is vesicular, of the size of pemphigus, filled with a sero-sanguinolent fluid. Nearly all the eruptions had a dark purple color at the base, reminding one of syphilide. New pustules and vesicles had continued to appear from time to time, but were all located on the vaccinated arm between the shoulder and the elbow, except a single pustule which had appeared on the opposite arm. This patient denied ever having had syphilis in any form; neither had he been subject to cutaneous eruptions. None of the patients to whom I have alluded here, were vaccinated by myself, and I know nothing of the history of the vaccine matter used. From the reports and rumors on this subject, I think the bad results here alluded to have been sufficiently numerous to materially increase the prejudices against vaccination, in the minds of many of our citizens.

Such results can only be accounted for in three ways: First, by supposing the vaccine virus to have been taken from persons who were previously infected with syphilis or some other infectious disease. Second, by supposing that the vaccine virus, though from pure and healthy subjects, had been so used as to undergo a putrefactive change, by which it had acquired the properties of a purulent infection. Might not this occur either from allowing the scab to be put up *too* moist, or still more by repeatedly, at different times, wetting and using from the same virus kept between two plates of glass. In this way, virus that had been productive of perfect results with many of the past cases vaccinated, might, in subsequent ones, prove bad. Third, by supposing the existence, in the individual vaccinated, of some latent constitutional disease, which the vaccine only serves to excite into activity. This is, doubtless, the true explanation of a large number of the cases.

The second error to which I have alluded, consists in a general neglect to examine the cases vaccinated at suitable intervals during the progress of the vaccine. Hundreds of those vaccinated are never examined after the operation is performed. If all the profession would make it a rule to examine carefully the vesicular stage of the vaccine sores, very many spurious cases would be detected and remedied, and some bad results, by a little treatment, would be greatly mitigated. Everything relating to vaccination should receive more systematic attention from the profession than is usually given to it.

Rubeola, or measles, has also continued unusually prevalent during the last four months. And, among the poorer classes especially, pneumonia has been a very frequent complication or sequel. In young children, the pneumonia has supervened insidiously in the lobular form, and proved fatal in a large proportion of the cases. From my own observations, I think the fatality has been greatly increased from the two following causes: First, a large proportion of the community no sooner suppose the child to have measles than they wrap it in thick blankets, keep the room inordinately hot, stop all ventilation, and industriously ply it with hot stimulating drinks, for the purpose of getting out and keeping out the eruption. A more cruel or injurious practice could not readily be devised. Second, during the active progress of the measles, the pneumonic or broncho-pneumonic symptoms are not distinguished from the ordinary catarrhal symptoms that belong to the eruptive fever, and hence no danger is apprehended, and in many instances no physician is called until the latter has completed its course and the patient is found still becoming worse instead of recovering. The physician now finds the lungs hepatized and the vitality of the little sufferer so far exhausted that no remedies will avail. An examination of the monthly bills of mortality shows the following results: from measles, in October, 4; November, 8; January, 21; February, 24; March, 21. From pneumonia, in October, 11; November, 19; January, 49; February, 54; March, 28. These figures are much above the average from these diseases. Thus in the same months of 1866 and 1867, the returns were as follows: from measles, in October, 6; November, 1; January, 0; February, 0; March, 1. From pneumonia, in October, 3; November, 13; January, 11; February, 20; March, 12.

In relation to the prevalence of other diseases, we have observed nothing requiring special mention. There have been occasional cases of scarlet fever and diphtheria, and the usual prevalence of catarrhal and rheumatic affections. During the latter part of the continuous cold weather of January, attacks of meningitis, convulsions, and croup were more frequent. During the months of February and March, I have met with cases of diarrhœa and dysentery more frequently than is usual in those months. These, together with the peculiarly oppressive qualities of the atmosphere during the two or three warm days that occurred in the first starting of the frost and ice in the last of February, have caused me to look with some anxiety in reference to a return of epidemic cholera the coming summer.

The weekly and monthly reports of the Board of Health have led many to suppose that the past year has been unusually healthy in this city. This has been done by comparing the mortality of each month in 1867 with the corresponding month in 1866. The year 1866, as is well known, was characterized by a special epidemic of cholera, which added nearly 1000 to the gross mortality of that year, and hence does not afford a proper standard of comparison in reference to the ordinary ratio of mortality. If we compare the ratio of mortality in 1867 with that of 1865, when no special epidemic was prevalent, it will afford a much better test and show very different results. Thus, the aggregate mortality for 1865 was 3663, the population 185,000, making the ratio 1 death to 50 of the population. The aggregate mortality of 1867 was 4604, the population 230,000, making the ratio 1 death to 49 of the population. This will be found fully up to the average ratio for the last 10 years.

In respect to the sanitary condition of the South Division of the city, which I represent, and, indeed, of all the Divisions of the city, there are visible simply the following improvements over past years, namely: fewer heaps of ashes and garbage, especially in the central parts of the city, and a somewhat less number of heaps of stable manure. The institution of a scavenger system, imperfect and irregular as it is, has materially lessened the winter accumulations of the kind just named; still manure heaps of respectable age are abundant in all parts of the city. Full out-door privies are still more abundant; and even ditches obstructed by both ashes and garbage are not wanting. The point of vital importance to the sanitary welfare of our city, however, seems to be almost overlooked or neglected. I allude to the overflow and thorough saturation of the soil during the spring, with water holding in solution all the products derived from macerating manure heaps, contents of privies, etc., and disappearing chiefly by evaporation so as to leave in the soil all the animal and vegetable matter previously held in solution, in a condition admirably adapted to undergo decomposition and evolve into the air a variety of actively poisonous products so soon as the higher temperature of summer is brought to bear on it. Were it not for this source of mischief, our wide streets swept freely by the winds from the lake and the prairie, would render Chicago one of the healthiest cities in our country. There are, however, at this time (April 1st), notwithstanding all the improvements made during the past year, whole sections of the city, closely inhabited, the soil of which is completely saturated and partially covered with standing water, and over which are scattered a sufficient number of manure heaps and open privies to thoroughly impregnate the whole with both animal and vegetable matter. During the succeeding two months of cool weather, the excess of water will evaporate, leaving the animal and vegetable residue in precisely such a condition as is most favorable for the evolution of miasms, sporules, and fungi under the high temperature of July and August.

It is in vain to expect any marked or permanent improvement in the health of the city while such a state of things exists. The removal of ashes and garbage, and the sweeping of a few central streets, is all proper and necessary; but it has comparatively little effect on the health of the citizens. There may be some who think the great evil of stagnant surface water, in large parts of the city, is unavoidable, on account of the low and level character of the surface. This is true, however, only to a limited extent; for the larger number of the very worst places are fully accessible to surface drainage into good permanent sewers already constructed. Take, for instance, that part of the North Division lying between North Market Street and the North Branch of the river, and south of Division Street, large portions of which are now either overflowed or fully saturated with standing water. The middle and rear part of the lots fronting directly on sewered streets are, in very many instances, so covered with water that the stable and the privy are like islands, accessible only by planks or boards. And yet nothing is wanting, to relieve the whole, except one or two hours of labor in opening suitable channels to the street gutter.

In visiting some patients on Townsend, Bremer, and Wesson Streets two or three days since, I found the rear part of nearly all the lots fronting on these streets saturated with stagnant water and thoroughly impregnated with the contents of privies and stable refuse, and the street gutters full. On further examination, I found nearly all the inlets of the Chicago Avenue and other sewers in that region more or less obstructed. Six good laborers armed with hoe, spade, and axe, under the direction of a man of intelligence and common-sense, could have relieved the whole of that territory of its surface water in two days, and left it in such a condition that the subsequent showers of spring would have washed and aided in purifying the surface instead of macerating it for weeks, and returning into the air loaded with miasms.

In visiting a patient yesterday, at 605 West Lake Street, a few rods west of Union Park, I had occasion to pass the rear end of the lot. I found, not only the rear end of that lot, but the alley, and as far as I could see along the adjoining lots, more or less covered with standing water well impregnated with animal and vegetable matters. Yet, in the centre of Lake Street, directly in front of the whole block, was a good brick sewer, on such a level as to be capable of draining the whole, if there had been any side gutters leading into it. Scores of similar instances might be pointed out in the south and southwest parts of the city, but these are sufficient for illustration.

My principal object in calling the attention of the Society to this subject, is to urge the exertion of its entire influence in accomplishing the two following items:

First. The adoption by the Boards of Health and of Public Works, of proper ordinances requiring the owner or occupant of every lot in the city, to so connect its surface with the adjoining street gutters that all water falling upon any part of such surface shall have an unobstructed access to such street gutters.

Second. The adoption and rigid enforcement of a rule, that on the first breaking up of winter, which is generally the last week in February or the first in March, a sufficient force of laborers, under the direction of a suitable number of intelligent overseers, shall be employed to clear all obstructions, of whatever kind, from the entrances to the sewers, and from the street and alley and lot gutters.

By having the work done promptly, *at the time indicated*, it would not only prevent the long retention of the water belonging to the first spring freshet, impregnated with the accumulations of winter, but it would render every subsequent spring shower a cleansing and purifying agent. It would cost no more to employ a sufficient number of men to do all the required work in ten days, at the exact season when it is of the highest degree of sanitary importance, than it would to keep a small number at work during the whole season, and have three-quarters of the work done so late in the season as to lose nearly all its value, in a sanitary aspect.

If our Board of Health shall continue much longer to keep a large number of sub-officials employed in making sanitary inspections, and gathering statistics to cumber the shelves of its office, and leave the most important part of all its sanitary work undone, or done so late in the season as to be of little practical value, it is certain that the great mass of tax-payers will soon begin to regard the whole machinery as a useless pecuniary burden. Every practising physician should feel an interest in maintaining an efficient system of sanitary regulations, and the accurate registry of statistics of births, marriages, and deaths. And it is from no desire to find fault with the present Board of Health, that I have called your attention to the foregoing topics, but from an ardent desire to aid in rendering its work more efficient and timely in the prevention of sickness and death.

